# Metabolism heterogeneity in melanoma fuels deactivation of immunotherapy: Predict before protect

**DOI:** 10.3389/fonc.2022.1046102

**Published:** 2022-12-22

**Authors:** Xinyue Zhang, Zongguang Tai, Fengze Miao, Hao Huang, Quangang Zhu, Leilei Bao, Zhongjian Chen

**Affiliations:** ^1^ Shanghai Skin Disease Hospital, School of Medicine, Tongji University, Shanghai, China; ^2^ Department of Pharmacy, Third Affiliated Hospital of Naval Medical University, Shanghai, China; ^3^ Department of Pharmacy, Jiangxi University of Chinese Medicine, Nanchang, China

**Keywords:** melanoma, immunotherapy, metabolism heterogeneity, tumor microenvironment, combination therapy

## Abstract

Malignant melanoma is widely acknowledged as the most lethal skin malignancy. The metabolic reprogramming in melanoma leads to alterations in glycolysis and oxidative phosphorylation (OXPHOS), forming a hypoxic, glucose-deficient and acidic tumor microenvironment which inhibits the function of immune cells, resulting in a low response rate to immunotherapy. Therefore, improving the tumor microenvironment by regulating the metabolism can be used to improve the efficacy of immunotherapy. However, the tumor microenvironment (TME) and the metabolism of malignant melanoma are highly heterogeneous. Therefore, understanding and predicting how melanoma regulates metabolism is important to improve the local immune microenvironment of the tumor, and metabolism regulators are expected to increase treatment efficacy in combination with immunotherapy. This article reviews the energy metabolism in melanoma and its regulation and prediction, the integration of immunotherapy and metabolism regulators, and provides a comprehensive overview of future research focal points in this field and their potential application in clinical treatment.

## 1 Introduction

In 2020, 325,000 new cases of melanoma were reported worldwide with 57, 000 deaths (1). Melanoma, a skin-derived malignancy, is the highest mortality-associated skin disease and one of the tumors with the fastest growing incidence (2).

**Figure 1:**
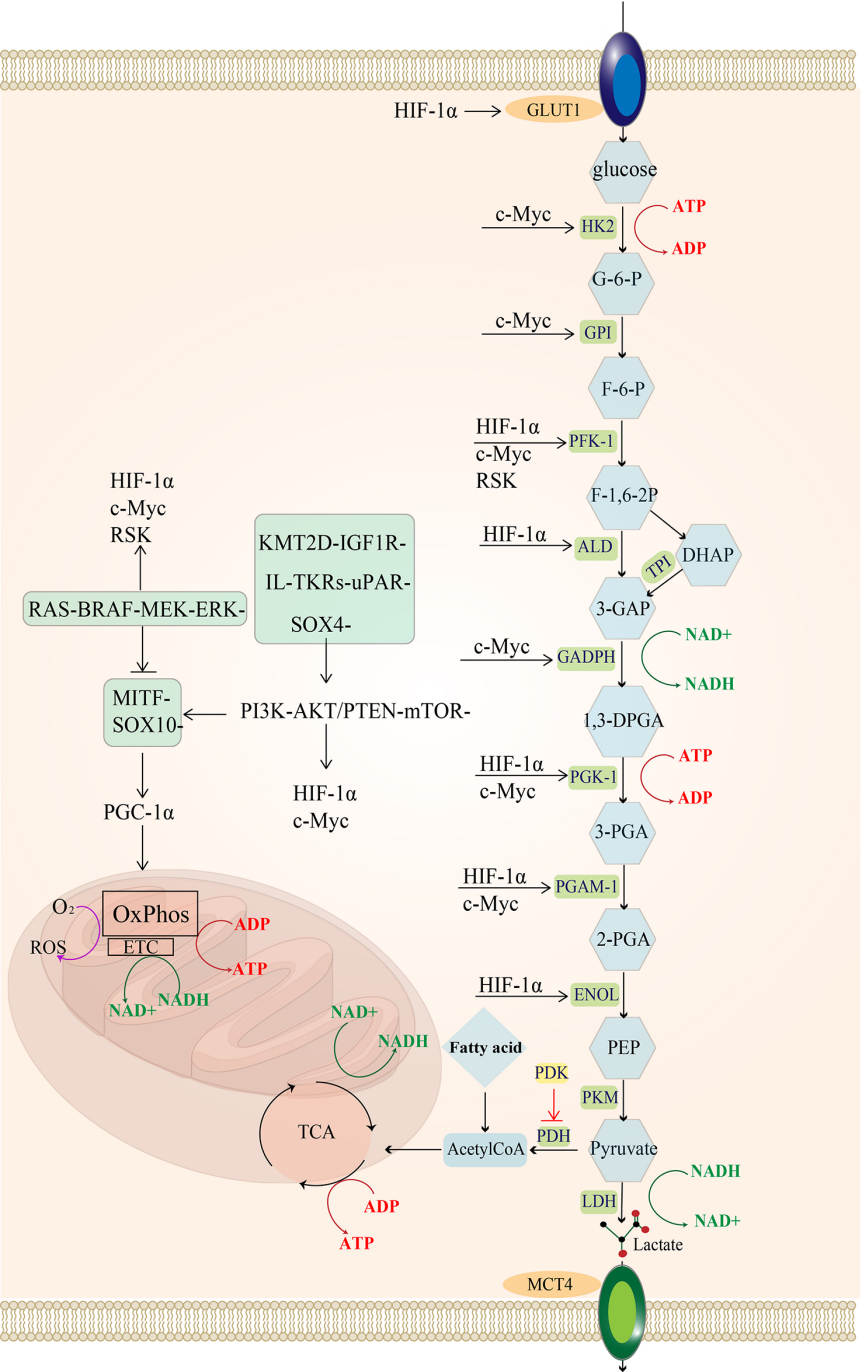
Regulatory pathways of energy metabolism in melanoma: MAPK/RAS pathway and PI3K pathway activate downstream MYC and HIF-1a. In melanoma, the urokinase-type fibrinogen activator receptor (uPAR) is a major factor in the fibrinolytic system and induces a decrease in the MAPK/p38 activity ratio, leading to tumor proliferation and consequently to tumorigenesis and even metastasis (5). uPAR coupled to the integral protein-linked tyrosine kinase receptor IL-TKRs induces the PI3K/pAKT/mTOR/HIF-1α pathway (6), promoting the Warburg effect of melanoma and increasing glycolysis (7). KMT2D is a histone H3 lysine 4 (H3K4) methyltransferase, and H3K4 methylation reprogramming has an important role in BRAFV600E melanogenesis. KMT2D-deficient tumors have been reported to show substantial reprogramming of metabolism, such as the IGF1R-PI3K-AKT pathway, which activates the downstream HIF- 1a and Myc, leading to the upregulation of glycolysis (8). uPAR and the KMT2D pathway can activate the PI3K pathway. The MITF pathway is blocked by MAPK and can also be activated by mTOR and the SOX-10 transcription factor, suppressing HIF-1a expression. Importantly, the SOX-2 transcription factor can directly suppress HIF-1a. glucose transporter 1 (GLUT-1); hexokinase 2 (HK-2); glucose 6-phosphate (G-6-P); glucose hexose isomerase (GPI); fructose 6-phosphate (F-6-P); phosphofructokinase 1 (PFK-1); 1,6 fructose diphosphate (F-1,6-BP); aldolase (ALDOA); 3-phosphoglyceraldehyde (3-GAP); Dihydroxyacetone phosphate (DHAP); Triose-phosphateisomerase (TPI); 3-phosphoglycerol dehydrogenase (GADPH); Coenzyme I (NAD+);1,3-diphosphoglycerate phosphoglycerate kinase (PGK); 3-Phosphoglyceric acid (3-PGA); adenosine diphosphate (ADP); phosphoglycerol isomerase 1 (PGAM-1); 2-phosphoglyceric acid (2-PGA); enolase (ENOL); Phosphoenolpyruvate (PEP); pyruvate kinase (PKM); lactate dehydrogenase (LDH); monocarboxylate transporter protein 4 (MCT-4); tricarboxylic acid cycle (TCA); pyruvate dehydrogenase (PDH).

Melanoma exhibits distinct biosynthesis and energy metabolism that can accelerate its proliferation, promote metastasis, and eventually leads to drug resistance and ineffectiveness of treatment (2). Over the past decade, tumor cells have been established to promote their growth by reprogramming their energy metabolism (3). The Warburg effect provided the initial clue that tumor cells depended more on glycolytic reactions for energy production and reduced mitochondrial oxidative phosphorylation than normal cells (4). Glycolysis and oxidative phosphorylation have become a research hotspot in tumor metabolism since then. Glycolysis is a metabolic process whereby cells rapidly produce adenosine triphosphate (ATP) (**Figure 1**), with one molecule of glucose producing 2 ATP while three carbon atoms turn into lactate. Mitochondrial oxidation is a slow ATP-producing reaction, with one molecule of glucose producing 28 ATP (10 ATP from the tricarboxylic acid cycle and 18 ATP from oxidative phosphorylation). Therefore, glycolysis consumes more glucose to produce equal ATP. Nonetheless, unused carbon can be used for growth and proliferation through the cellular carbon metabolic pathway to satisfy the strong proliferation demand. Therefore, glycolysis (anaerobic glycolysis) is more suitable for rapidly proliferating cells than mitochondrial oxidation (9).

However, contrasting studies suggest that glycolysis and oxidative phosphorylation can be simultaneously upregulated in melanoma based on the metabolic demands of the tumor. These studies draw our attention to the metabolic heterogeneity in melanoma. Due to the heterogeneity of tumor vascular and intratumoral structures, the nutrient and oxygen contents in different parts of the tumor may fluctuate, and the tumor cells can flexibly change their metabolism according to their own energy requirements and the nutrient supply of the tumor microenvironment, which is called metabolic heterogeneity (10, 11).. Therefore, the flux of glycolysis and mitochondrial oxidative phosphorylation exhibit differential regulation in melanoma and can reflect the state of the tumor cells to some extent. Overall, understanding the growth status of melanoma *via* its energy metabolism for application in targeted therapy may be a potential solution for melanoma patients (12–14).

However, the high energy metabolic demand of melanoma leads to local glucose deficiency and lactate accumulation in the tumor microenvironment. This type of TME inhibits T cell activation and recruits immunosuppressive cells, such as tumor-associated macrophage (TAM), regulatory (Treg) cells and myeloid-derived suppressor cells (MDSCs) (15), which models the immunosuppressive tumor microenvironment, and is one of the reasons why immune checkpoint inhibitors are ineffective in the treatment of melanoma (16, 17).

The interaction between the immune regulation and energy metabolism of melanoma has triggered interest in a synergistic approach involving immunotherapy and metabolic therapy. Regulating the tumor immune microenvironment based on the metabolic characteristics of melanoma may be a potential method to improve immunotherapy’s efficacy and achieve synergistic treatment goals (18). This article will review the metabolic characteristics of melanoma and its regulation, present an overview of the mechanism of local immunosuppression and introduce the biomarkers reflecting the tumor metabolism to provide new insights for targeted treatment through the combination of energy metabolism regulators and immunotherapy.

## 2 Metabolic heterogeneity

Altered amino acid metabolism such as glutamic acid and alanine would accelerate melanoma proliferation; up-regulated fatty acid oxidation boosts melanoma to gain resistance to therapy. Nonetheless, the alteration of glycolysis and oxidative phosphorylation cover both. In melanoma, glucose metabolic alterations are high plasticity, and highly associated with metastasis. Hence, an insight into the mechanisms of glucose metabolic dysregulation of melanoma is necessary (19, 20).

### 2.1 Signal pathways of metabolic heterogeneity

Major drivers of melanomagenesis include the activation of NRAS/BRAF or the loss of Phosphatase tensin homologue (PTEN) or cyclin dependent kinase inhibitor 2A (CDKN2A) (21). Among them, BRAF variations occur in 66% of melanomas (22), which can lead to metabolic reprogramming (23).The most frequent BRAF mutation is BRAFV600E, where the 600^th^ valine position of the BRAF mutation is replaced by glutamate (BRAFV600E), leading to constitutive activation of its serine/threonine kinase activity (24). It has been established that BRAFV600E mainly affects glycolytic flux. PTEN, a tumor suppressor gene, synergistic altered of a metabolic pathway with BRAF mutations (25). The protein encoded by PTEN affects the PI3K/AKT/mTOR signaling pathway activation and downstream metabolism-related pathway (26, 27). CDKN2A regulates mitochondrial function to induce melanoma (28).

Additionally, Microphthalmia-associated transcription factor (MITF) is also expressed in 10%–20% of human melanomas, which induce pigmentation thereby product melanin (29). MITF is upregulated by the PI3K pathway and downregulated by the MAPK pathway (30, 31). Current evidence suggests that MITF is closely related to mitochondrial energy metabolism in melanoma (32–34).

#### 2.1.1 Up-regulation of Glycolytic

The MAPK (RAF-MEK-ERK) signaling pathway is frequently activated in melanoma (35), and it can phosphorylate the downstream p90 ribosomal S6 kinase (RSK) (36), which activates fructose-2,6-bisphosphatase 2 (PFKFB2), an isoform of phosphofructokinase, increasing fructose-2,6-bisphosphate synthesis (a glycolytic intermediate) (37). Additionally, RSK phosphorylates fructose-2,6-bisphosphatase 1-4 (PFKFB1-4), an isoform of phosphofructokinase, to upregulate phosphofructokinase 1 (PFK-1), a significant rate-limiting enzyme of glycolysis, which ultimately increases glycolytic flux (38, 39).The hypoxia-inducible factor (HIF1α) is a regulator of glycolysis, and its production is promoted by the activation of PI3K-PTEN/AKT and MAPK signaling pathways (40–42). The MAPK signaling pathway can inhibit the MITF-PGC1α axis, and the PI3K-PTEN/AKT signaling pathway indirectly stimulates the mammalian target of rapamycin (mTOR) protein. Both can increase downstream HIF-1α activity (43). Moreover, increased HIF-1α activity promotes the expression of enzymes involved in glycolysis, such as PFK, aldolase (ALD), 1,3-diphosphoglycerate phosphoglycerate kinase 1 (PGK1), enolase (ENOL) (44), as well as glucose transporter 1 (GLUT-1,GLUT-1 is one of the GLUT family of membrane transport proteins dedicated to intracellular glucose uptake) (45), ultimately increasing glucose uptake and glycolysis flux.

Additionally, HIF-1α can limit OXPHOS in mitochondria by regulating the expression of PDK, an enzyme that phosphorylates and inactivates pyruvate dehydrogenase (PDH). It has been established that PDH converts pyruvate to acetyl coenzyme A (14) and promotes mitochondrial oxidation. In other words, the presence of HIF-1α in advanced melanoma suggests that melanoma is less dependent on oxidative phosphorylation and more on glycolysis for ATP production during hypoxia (46, 47).

Another crucial element influencing melanoma glycolysis is c-Myc (40), which is a member of the MYC proto-oncogene family (c-Myc, L-Myc, and N-Myc are collectively known as MYC) that encodes the helix-loop-helix leucine zipper (bHLHZip) transcription factor (48, 49). It has been shown that the c-Myc protein is regulated by the PI3K and MAPK pathways; c-myc induces the expression of almost all glycolytic enzyme genes, including HK, GPI, PFK-1, 3-phosphoglycerol dehydrogenase (GADPH), 1,3-diphosphoglycerate phosphoglycerate kinase (PGK) and ENOL (50).

High levels of c-Myc inhibit MondoA, a key downstream negative regulator of glucose uptake, and then indirectly increase glucose uptake and enhance glycolytic flux by inducing thioredoxin-interacting protein (TXNIP) and inhibiting GLUT1 expression (40).

Interestingly, c-Myc exerts different functionsdepending on the state of the tumor microenvironment (TME) (51). Unlike HIF1α, c-Myc mainly regulates oxygen-enriched tumor cells by upregulating aerobic glycolysis and mitochondrial activity since c-Myc directly promotes glycolysis and reduces pyruvate kinase 2(PKM2) activity (52), thereby limiting PKM2-mediated phosphoenolpyruvate-pyruvate conversion (i.e., the last irreversible step of glycolysis). These changes result in the accumulation of upstream glycolytic intermediates, while the increased carbon during glycolysis is used for mitochondrial biosynthesis processes, such as manufacturing fatty acids, amino acids and other substances required for proliferation (53–55).

Meanwhile, both c-Myc and HIF-1α promote the expression of lactate dehydrogenase A (LDHA), which encodes the production of the five isoforms of LDH when combined with lactate dehydrogenase B (LDHB). Lactate dehydrogenase (LDH) is a crucial enzyme in the utilization of lactate, and LDHA encodes lactate dehydrogenase 3-5(LDH3-5), which completes the Pasteur effect by catalyzing the conversion of pyruvate to lactate (56). During the conversion of pyruvate to lactate, LDH can reduce NADH to NAD+. The reduction product NAD+ is then used as an electron acceptor by the glycolytic enzyme GAPDH, which leads to the conversion of NAD+ to NADH, thus forming an intracellular NAD+ cycle (57, 58). Sustained high levels of glycolysis consume a large amount of NAD+, and the promotion of LDHA expression induced by c-Myc and HIF-1α directly increases the LDH content, which can supplement the NAD+ and ensure sustained glycolysis (**Figure 1**).

#### 2.1.2 Alteration of oxidative phosphorylation

High serum lactate levels in patients with melanoma are associated with increased glycolytic flux (59). However, many glycolysis inhibitors (e.g., 2-deoxyglucose) do not yield satisfactory inhibitory effects on tumor growth since glycolysis is not the only energy metabolic pathway in melanoma, and energy metabolism can be flexibly regulated based on the tumor microenvironment.

In addition to glycolysis (60), OXPHOS flux has been reported to be higher in metastatic melanoma cells cultured *in vitro* compared to melanocytes. Besides, OXPHOS is reportedly elevated in clinical stage IV melanoma (61). OXPHOS, the last reaction in cellular respiration, produces the most ATP by transporting electrons *via* proton complexes embedded in the inner mitochondrial membrane. Oxygen is necessary for OXPHOS, acting as the terminal electron acceptor in the electron transport chain. OXPHOS produces 90% of the ATP cells need in the presence of oxygen. Therefore, an increase in oxidative phosphorylation is associated with increased mitochondrial energy production (62), mitochondrial biosynthesis, and cell proliferation (63–65).

Deletion of CDKN2A is a common feature in melanoma (66), which leads to down-regulation of downstream p14^ARF^ expression. p14^ARF^ inactivates melanocyte mitochondria by interacting with B-cell lymphoma-extra large (BCL-X_L_), translocating to dysfunctional mitochondria and inducing loss of membrane potential (67). Whereas down-regulation of p14^ARF^ results in sustained activity of dysfunctional mitochondria with higher metabolic plasticity (23).Melanoma exhibits increased mitochondrial respiration, mainly related to MITF expression. As a specific transcription factor in melanoma cells, MITF is a key regulator and promoter of mitochondrial respiration (65, 68). MITF promotes mitochondrial respiratory gene transcription driven by downstream activation of peroxisome proliferators activated receptor gamma co-activator 1-alpha (PGC1α) (69, 70). PGC1α, a well-known peroxisome proliferator-activated receptor γ coactivator 1 (PGC-1) family member, is one of the first genes upregulated for increased mitochondrial biogenesis and OXPHOS (65, 66).

PGC-1α levels and mitochondrial quality heterogeneity is associated with transitions between proliferative and metastatic phenotypes of melanoma cells (71). It has been shown that PGC-1α increases the expression of antioxidant genes (72) and tolerance to the deleterious effects of mitochondrial oxidative respiration, enabling melanoma cells to survive under oxidative stress (65, 72, 73). In this respect, during electron transfer in the mitochondrial respiratory complex, a small fraction of oxygen undergoes incomplete reduction and becomes reactive oxygen species (ROS) (74). At low ROS concentrations, genomic instability is induced, and cell proliferation is stimulated, while at high ROS concentrations, ROS result in oxidative stress cytotoxicity (75). As mitochondria continuously produce ROS, tumor cells exhibit the ability to detoxify ROS by expressing PGC1α (72) to regulate mitochondrial ROS production (76) and to increase intracellular ROS-consuming proteins, such as SCARA3 (77).

What’s more, the inhibition of oxidative metabolism by BRAF in melanoma is an independent process. For instance, the reduction of cytochrome oxidase subunit expression *via* HIF-1α (78, 79) does not affect the PI3K-mTOR pathway promoting the transcription of MITF-PGC1α and OXPHOS-related genes (**Figure 1**).

Thus, the response of PGC1α to MITF is the main regulatory factor of mitochondrial respiration in melanoma. The expression of MITF leads to the upregulation of PGC-1α (80), which promotes mitochondrial oxidative respiration and reduces its detrimental effects, which underlie melanoma’s recurrence and metastasis (81).

#### 2.1.3 The interaction of genetic and metabolic heterogeneity

Tumor heterogeneity is the terminology used to indicate that there are subclones of tumor cells with distinct molecular variations in one patient. Like many other tumors, melanoma is highly genetically heterogeneous (82), highlighting that different melanoma cells have diverse and unstable genetic backgrounds. It has been established that genetic heterogeneity underlies metabolic heterogeneity (83). Genetic pathways can regulate glycolysis as well as oxidative phosphorylation. In turn, metabolic heterogeneity can affect genetic heterogeneity, and the tumor microenvironment shaped by various metabolic patterns can induce the corresponding gene expression.

The Sex-determining region Y-related high mobility group-box (SOX) family is a typical example. The SOX gene family has been reported to play an important role in melanoma development and progression. SOX10 is essential for melanocytes and highly expressed in melanoma (84); it directly binds to the MITF promoter to upregulate MITF expression and activates PGC-1α to promote mitochondrial oxidative phosphorylation (85). Moreover, overexpression of SOX4 in melanoma leads to phosphorylation of AKT and activation of downstream HIF-1α and c-Myc to promote glycolysis in tumor cells (86). Meanwhile, SOX4 promotes OXPHOS by increasing the expression of MITF (87), which is not always antagonistic to glycolysis, allowing for a more flexible metabolism of tumor cells (65).

It has been shown that melanoma cells with high glycolytic flux accumulate lactate, resulting in an acidic environment that enhances SOX2 expression. When SOX2 protein expression exceeds HIF1α, SOX2 can directly bind to the HIF1α promoter to reduce HIF1α activity, thereby inhibiting glycolysis and promoting mitochondrial oxidative respiration *via* PGC-1α (88). When SOX2 is silenced, the metabolic phenotype of the cells switches back to a glycolytic-dominant mode. Thus, SOX2 expression prevents cell damage induced by excessive glycolytic acidification of the tumor microenvironment (45, 89).

Overall, the interaction between the SOX family and metabolic modalities reflects the metabolic flexibility of melanoma (45, 90, 91). Glycolysis and mitochondrial respiration, the two major energy production pathways in tumor cells, are activated to varying extents based on genetic backgrounds, reflecting the metabolic heterogeneity of melanoma (73, 92–94).

### 2.2 Molecules reflecting metabolic heterogeneity

#### 2.2.1 MCT

Excessive lactate concentrations in melanoma lead to growth arrest (95), and lactate efflux is an important mechanism for maintaining cellular glycolysis levels and preventing oxidative stress (96–98). Since lactate in tumor cells is transported by a family of monocarboxylate transporters (MCT), HIF-mediated upregulation of MCT is essential to prevent intracellular acidification (99). Interestingly, monocarboxylate transporter 4(MCT4) transfers lactate produced by glycolysis to TME, while monocarboxylate transporter 1(MCT1) transports lactate from TME to tumor cells.

Upregulated MCT4 expression has been associated with poor prognosis (100). Elevated MCT4 promotes lactate export to produce an immunosuppressive microenvironment (99, 101), and the excretion of H+ ions help to unblock its inhibitory effect on glycolytic enzymes (98, 102). Therefore, elevated MCT4 expression indicates that cells adopt glycolysis-based metabolism.

In metastatic melanoma, the expression of MCT1 is often upregulated, and the lactate transported into melanoma cells through MCT1 is converted into pyruvate for mitochondrial oxidative respiration (53, 103). Therefore, lactate does not accumulate or damage cells. Recent research reveals that increased MCT1 expression indicates the vulnerability of tumor cells to mitochondrial bioenergetics (98). However, MCT1 expression in melanoma metastases is reportedly correlated with the primary tumor size (100).

#### 2.2.2 LDH

The expression of LDHA and LDHB is determined by the oxygen content of the tumor microenvironment, representing the glycolysis and oxidative phosphorylation levels, respectively. LDHA gene expression is increased under hypoxia; LDH3-5 catalyze pyruvate to lactate and promote glycolysis. LDHB encodes lactate dehydrogenase 1-2 (LDH1-2), which catalyze the conversion of lactate to pyruvate. The pyruvate supply providing the substrate for mitochondrial respiration and promoting oxidative phosphorylation (61). Therefore, LDHA and LDHB expression reflects the energy metabolism mode in tumors.

#### 2.2.3 PGC-1α

Melanoma cells expressing high or low levels of PGC-1 represent different phenotypic subgroups of energy metabolism (76). High levels of PGC1α are associated with a poorer prognosis in metastatic melanoma (65, 76) since melanoma with high PGC-1α expression has a higher mitochondrial biosynthetic capacity (71, 104). Therefore, high-PGC-1α-expressing melanoma depends more on OXPHOS for ATP and can also survive under oxidative stress conditions (65, 73, 94). A study found that low-PGC-1α-expression melanoma exhibited downregulated mitochondrial oxidative respiration (71) and was more dependent on glycolytic metabolism, which reduced ATP production but metabolized more glucose (105). Despite the high sensitivity of low-PGC-1α-expression melanoma to ROS-induced apoptosis, this cell subgroup exhibits a higher expression of pro-metastatic genes, including integrins, transforming growth factor β (TGFβ) and Wnt (71, 76, 106).

In summary, melanoma cells with high levels of PGC-1α produce ATP mainly through OXPHOS and have a stronger ability to proliferate (71), while those with low levels of PGC-1α are more dependent on glycolysis (76) and show greater metastatic capacity. However, Grant et al. found that melanoma brain metastases depended more on OXPHOS (11) (in contrast with melanoma lung metastases and primary melanoma). Although it may seem contradictory, it is widely thought that the predominant metabolic mode of metastatic melanoma may depend on the local tumor microenvironment (e.g., intracranial or extracranial melanoma metastases).

### 2.3 Metabolic symbiosis: The result of metabolic heterogeneity

**Figure 2:**
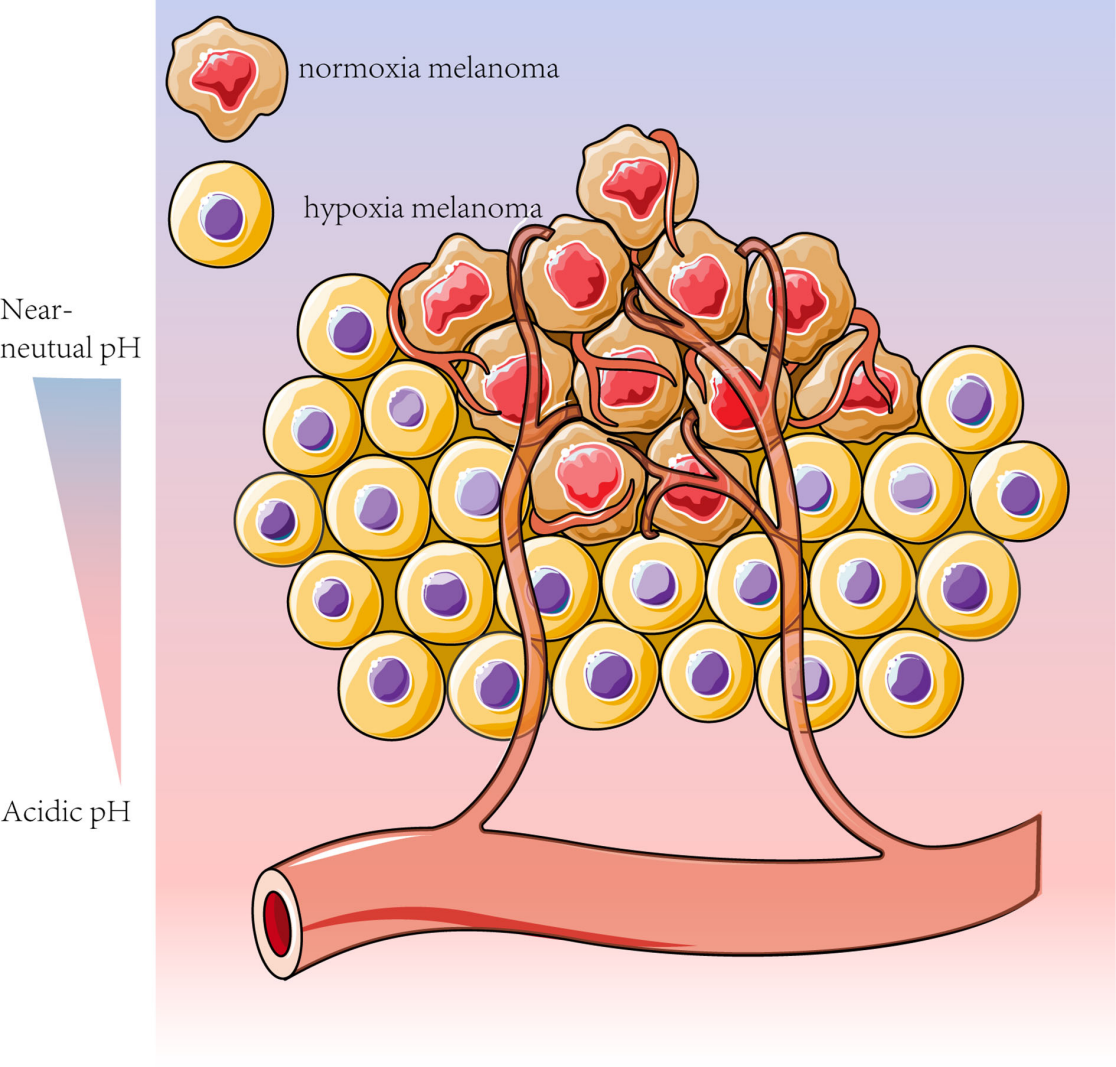
Melanoma cells can adapt their metabolism to the tumor microenvironment conditions, such as pH and oxygen content. To meet the high metabolic “demand” associated with melanoma dormancy, homeostasis and escape, the less oxygenated sites rely mainly on glycolysis, whereas the more vascularized tumors are more contingent on OXPHOS for energy production (14, 61). Based on this metabolic feature, melanoma cells can be divided into cells with enhanced glycolysis and inhibited OXPHOS under hypoxic conditions and other cells with adequate oxygen levels more dependent on OXPHOS (14).

Both glycolysis and OXPHOS are reportedly upregulated in advanced melanoma. Tumor cells in the hypoxic microenvironment rely mainly on glycolysis, while the remaining melanoma cells can take up the glycolytic product lactate and convert it to pyruvate *via* LDH. This phenomenon provides the substrate for mitochondrial oxidative respiration (61) and prevents lactate accumulation in hypoxic regions (91), which is known as metabolic symbiosis (14, 101). Melanoma cells in different regions have different metabolic patterns, and melanoma cells with different metabolic patterns can feed each other, providing sufficient metabolic substrates and a suitable environmental pH (**Figure 2**) (107, 108).

Various factors can contribute to the metabolic heterogeneity of melanoma, including oncogenes, tumor stage, intra-tumor vascular structure, and oxygen and glucose levels (100, 109, 110). This spatial and temporal metabolic heterogeneity promotes melanoma development, resulting in poor patient prognosis.

## 3 Metabolic heterogeneity and immune checkpoint blockade

An increasing body of evidence suggests that the activation of the PI3K pathway and overexpression of MYC (111) and HIF1α promote glycolysis and programmed cell death protein-1 ligand (PD-L1) expression (112, 113). Therefore, increased glycolytic flux indirectly implies increased PD-L1 expression, making melanoma relatively sensitive to programmed cell death protein-1 (PD-1) or its ligand PD-L1 blockade (114). However, a sustained high glycolytic flux causes an acidic and nutrient-limited immunosuppressive microenvironment, which leads to poor immunotherapy efficacy (115–117).

Hypoxia in the tumor microenvironment can impair immune cell functions, resulting from OXPHOS (113). In addition, gene pathways inducing oxidative phosphorylation do not overlap with those inducing PD-L1. Therefore, melanoma with high oxidative phosphorylation flux exhibits less PD-L1 expression and is less sensitive to immune checkpoint blockade.

It is well established that the metabolism of melanoma can result in an immunosuppressive tumor microenvironment. Moreover, the metabolic heterogeneity of melanoma will inevitably affect the metabolism of infiltrating immune cells at the tumor site (**Table 1**). This leads to the heterogeneous sensitivity the tumor to immune checkpoint inhibitors and is the reason for the lack of response to immune checkpoint therapy in some patients.

**Table 1:** Metabolism of immune cells in melanoma.

Immune cell	Infiltration site in the tumor	Oxygen-rich areas	Lactic acid enriched areas
**NK** 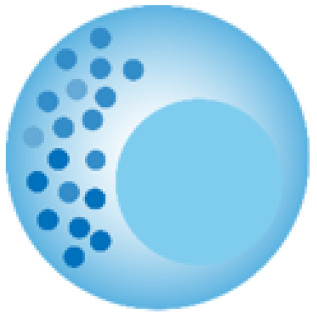	Mostly in the tumor cortex and at the lymph nodes (118)	glycolysis (119, 120)	Oxidative metabolism (118–120)
**M2** 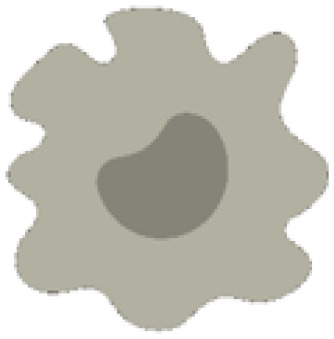	Accumulation in tumor areas with low oxygen levels and high lactate content (121, 122)	Oxidative metabolism (123)	Oxidative metabolism (123)
**DC** 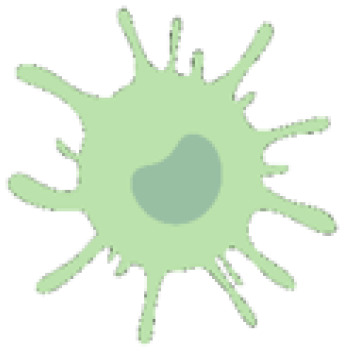	At the lymph nodes (124, 125)	Oxidative metabolism (126, 127)	Glycolysis (immature DC mostly) (128)
**Memory T cells** 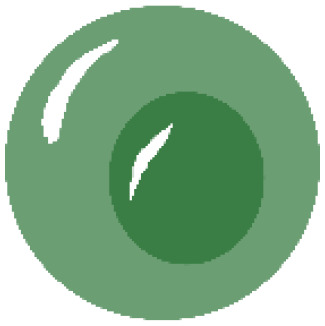	In perineural or lymph nodes (129)	Oxidative metabolism (55)	Oxidative metabolism (55)
**Treg** 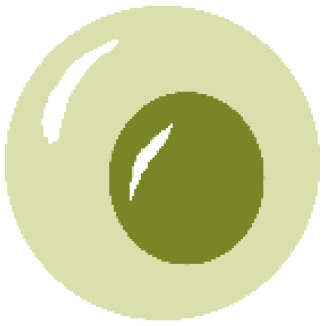	intra-tumor (130)	Oxidative metabolism (55, 131)	Oxidative metabolism (55, 131)
**T effector cells** 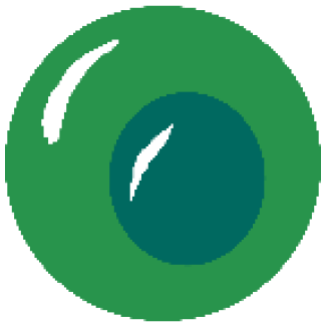	Accumulation in oxygen-enriched tumor areas (52, 126, 132–134)	glycolysis (135)	Inhibited glycolysis (135)
**M1** 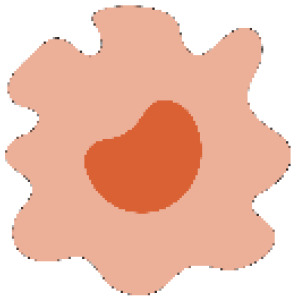	In oxygen-rich environment (122)	Glycolysis (123)	Inhibited glycolysis/conversion to M2 (123)

the metabolic heterogeneity of melanoma will inevitably affect the metabolism of infiltrating immune cells at the tumor site, which leads to heterogeneous sensitivity to immune checkpoint inhibitors, accounting for the lack of response to immune checkpoint therapy in some patients.

### 3.1 Glycolytic regions

Growing evidence suggests that melanoma cells in high glycolysis regions require high glucose consumption, leading to glucose deprivation in TME (119, 136, 137), which promotes the AMPK pathway in T cells, thereby inhibiting mTORC1 activity (mTORC1 and mTORC2 are the two distinct complexes of functional enzyme mTOR). As a result, T cell glycolysis is restrained (138). However, T cells require glycolysis for activation. Inhibition of glycolysis in T cells causes impaired T cell energy production and macromolecular synthesis (137, 139), leading to the failure to translate some mRNAs like interferon γ (IFN-γ) mRNA. IFN-γ is a pro-inflammatory cytokine produced by T cells that enhances the immune surveillance capacity of cytotoxic T lymphocytes (CTL) in TME (140). Therefore, melanoma limits T-cells function by glucose depletion.

**Figure 3:**
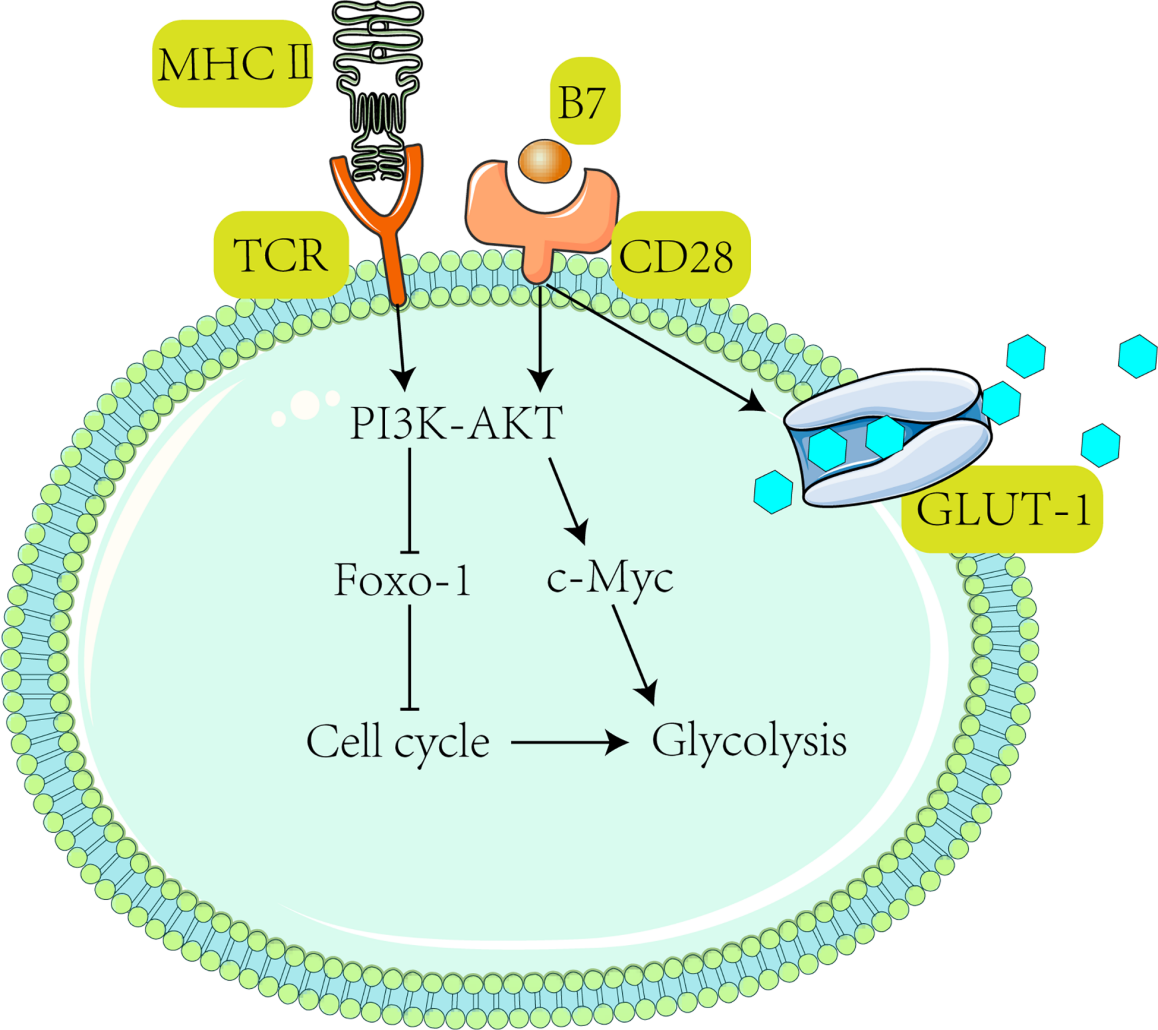
Activation of T cells requires receiving TCR and CD28-mediated co-stimulation, subsequently activating the PI3K-Akt pathway (47). After the PI3K-Akt pathway activates c-Myc, downstream Glut1 expression is stimulated to promote glycolysis (139) and T cell proliferation (146). It has been shown that activated T cells drive the PI3K-Akt-Foxo1 regulatory circuit (147, 148). Foxo1 (Forkhead box P1) is a transcriptional repressor of the T cell activation (147). Activation of AKT suppresses Foxo1 expression (149) and ultimately promotes T cell activation (47).

Moreover, acidity and lack of glucose in highly glycolytic melanoma can upregulate PD-1 expression in T cells (141, 142). In this respect, PD-1 can block the T-cell receptor (TCR)-mediated signaling pathway or PI3K pathway (143, 144), thus reducing glycolysis (45, 145) in T cells and inactivating them (**Figure 3**) (150).

**Figure 4:**
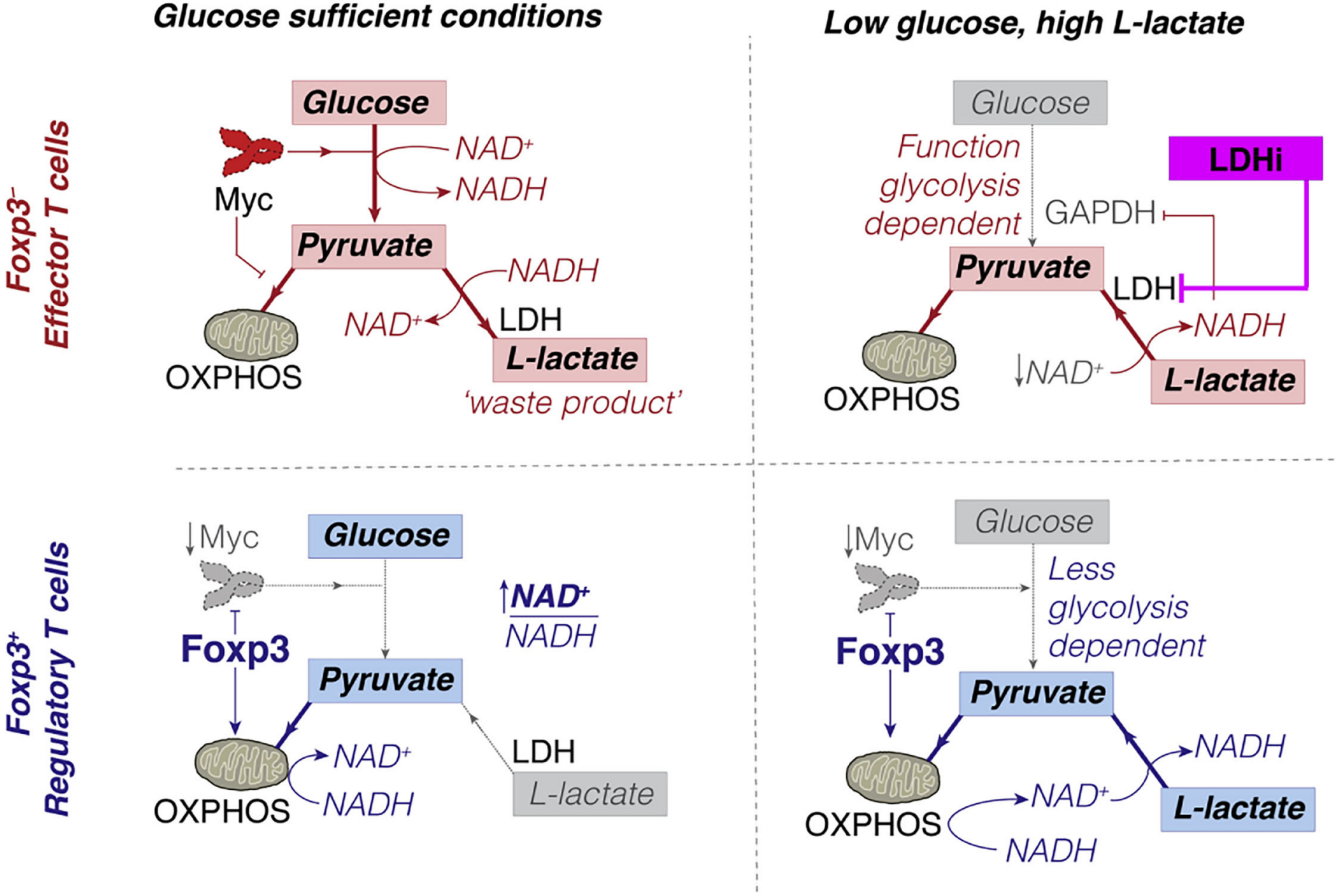
Conceptual model of how Foxp3+ Treg can escape the suppressive effects of low glucose, high L-lactate environments. Copyright ^©^ 2016 Elsevier Inc.

The decrease in CTL leads to an increase in the proportion of Treg in tumor microenvironments (52, 151, 152). Intriguingly, PD-1 does not restrain Treg metabolism (144, 153, 154) since Treg expresses forkhead box P3 (Foxp3); Foxp3+ Tregs inhibit glycolysis and promote OXPHOS (155, 156) (**Figure 4**), which both reduces NAD+ consumption (inhibits glycolysis) and increases NAD+ production (promotes OXPHOS). Therefore, Treg has more NAD+ for electron transfer, even when glycolysis is restricted (157). Tregs can take up lactate through MCT1 and convert it to pyruvate in the mitochondria for oxidative phosphorylation in a low-glucose tumor microenvironment.

However, it should be borne in mind that PD-1 is not always harmful to the immune system, given that PD-1 inhibits T cell glycolysis, which facilitates the formation of resting amnesic T cell populations (158). PD-1 stimulates T cells to reduce mitochondrial cristae and increase respiratory chain complexes (159). Accordingly, T cells have a substantial spare respiratory capacity (i.e., additional mitochondrial capacity for energy production in response to increased work or stress) (149, 160, 161). Glycolysis required in T cell activation can be replaced by mitochondrial oxidation (162–164), which contributes to the formation of memory T cells. Differentiation into memory T cells could prolong T cell survival (7, 165, 166) and preserve the potential for subsequent immunity. These changes may contribute to the response of melanoma to immune checkpoint blockade (ICB). However, PD-1 inhibits mTOR, which inhibits downstream PGC-1α, leading to increased mitochondrial oxidation and impairing the ability of T cells to alleviate oxidative stress (144).

Both glucose-restricted TME and PD-1 impair the immune effect of T cells. Although T cells may differentiate into memory T cells through metabolic transformation, this tumor environment can eventually inhibit cytotoxicity to tumor cells and induce immune quiescence (154, 165, 167).

T cells in high-glycolytic-flux melanoma areas express cytotoxic T lymphocyte-associated protein 4 (CTLA-4). Current evidence suggests that CTLA-4 downregulates CD28 and blocks all TCR signaling (168), inhibits AKT *via* protein phosphatase 2A (PP2A) (169), and downregulates T cell glycolysis (121). Moreover, inhibition of glycolysis induced by CTLA-4 does not promote mitochondrial oxidation, which avoids differentiation into memory T cells (143, 144). This phenomenon leads to the desensitization of T cells to tumor antigens and causes immune dysfunction (142).

In addition, lactic acid produced by glycolysis prevents the maturation of dendritic cells (DC), increases immunosuppressive cytokines such as IL-10 (121), and induces TAM polarization to an M2 phenotype (122). M2 releases vascular endothelial growth factor (VEGF) and indoleamine 2,3-dioxygenase (IDO), which promotes Treg migration to the tumor and inhibits natural killer (NK) cell function (122, 170, 171).

### 3.2 Oxidative phosphorylation region

**Figure 5:**
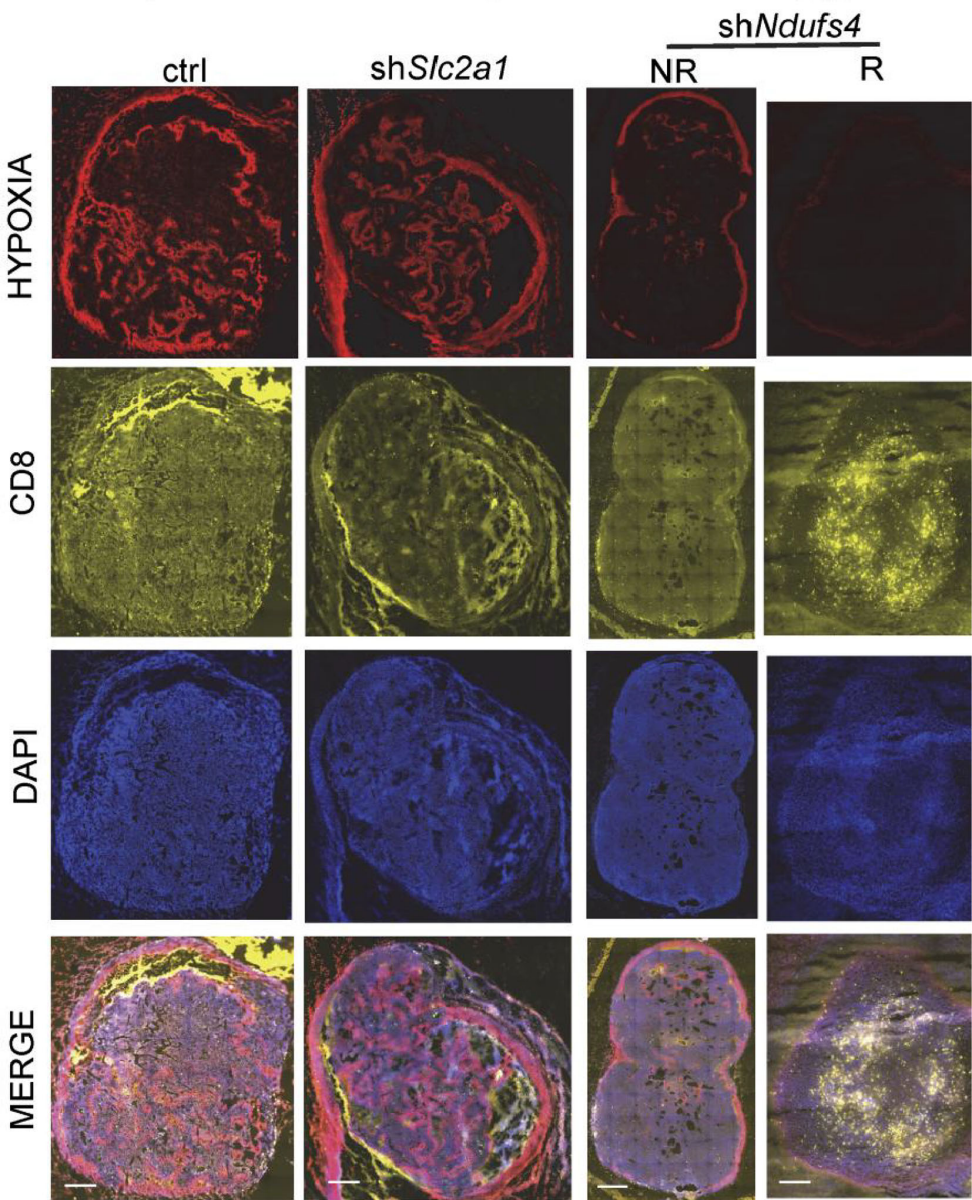
Hypoxia is present in the control group (elevated glycolysis, elevated oxidative phosphorylation) and the shSlc2a1 group (reduced glycolysis, elevated oxidative phosphorylation), while T cell accumulation in melanoma is low. The shNdufs4 group (glycolysis only) is less hypoxic, and the T-cell status is significantly improved. Copyright ^©^ 2019, American Society for Clinical Investigation.

T cell infiltration is a prerequisite for response to immunotherapy (172). Although CD8+ T cells in glycolytic regions are significantly reduced (**Figure 5**) compared to oxidative phosphorylation regions, the glycolysis-dominated melanoma does not compete with tumor-infiltrating lymphocytes (TILs) for oxygen. As a result, TILs are more fully metabolized and have higher cell mass despite reduced numbers (113). In contrast, although OXPHOS-dependent melanomas have more glucose, melanoma cells with high oxidative phosphorylation flux consume more oxygen, and the hypoxic microenvironment is more likely to cause T cell dysfunction (113), accounting for the shorter overall survival of patients with high oxidative phosphorylation flux than those with high glycolytic flux after PD-1 monoclonal antibody treatment. Therefore, targeting oxidative metabolism in melanoma is key to improving immunotherapy effects (113, 173).

The above phenomenon may be caused by the following reasons: (1) high oxidative phosphorylation flux induces hypoxia TME, which increases the expression of PD-L1 in melanoma and TAM (174). PD-L1 can recruit myeloid-derived suppressor cells to the tumor hypoxic region (175), thus forming an immunosuppressive microenvironmental barrier; (2) weak tolerance of T cells to hypoxia, causing T cell impairment or even failure; (3) decreased quantity and quality of T cell infiltration.

However, direct inhibition of mitochondrial oxidative phosphorylation leads to T-cell failure since TCR and CD28 co-stimulation promotes mitochondrial OXPHOS (176) and the activated mitochondria biosynthesize (177) as well as regulate T cell growth and proliferation (141, 163, 177, 178). Therefore, co-stimulation is required to avoid T-cell failure.

## 4 Immune checkpoint therapy with metabolic regulator

Since immune checkpoints are the primary cause of the progressive dysfunction of immune cells in TME (179), Jim Allison introduced the concept of ICB for cancer therapy in 1996 (180). In 2010, the CTLA-4 blocking antibody (Ipilimumab) became the first ICB treatment for metastatic melanoma (181). PD-1 is another T-cell immune checkpoint, and PD-1 or PD-L1 blocking antibodies were found to enhance control of a variety of tumors. Subsequently, PD-1 blocking antibodies (pembrolizumab and nivolumab) were approved for metastatic melanoma in 2014 (182). Given the effect of melanoma metabolism on immune cells, the combination of metabolic regulators and ICB therapy may be a promising approach (**Table 2**).

**Table 2:** Combinations of energy metabolism modulators with immunotherapy.

energy metabolism modulator	immunotherapy	Phase	NCT
**Metformin**	Nivoluvab/Pembrolizumab	Phase II	NCT04114136
**Metformin**	Pembrolizumab(KEYTRUDA ^®^)	Phase I/II	NCT03311308
**GSK2636771(Selective PI3K-Beta Inhibitor)**	Pembrolizumab	Phase I/II	NCT03131908
**GSK2636771(Selective PI3K-Beta Inhibitor)**	Trastuzumab/Pertuzumab/Nivolumab	Phase I/II	NCT02465060
**LDH inhibitor**	anti PD-1	Basic research (183)	
**IACS-010759 (OXPHOS Inhibitor)**	Anti CTLA4 and/or anti PD1	To be proven (11, 184)	
**diclofenac**	anti PD-1	Basic research (184)	
**Phenformin**	anti PD-1	Basic research (185)	
**Shikonin(PKM2 Inhibitor)**	Anti PD-L1	To be proven (186)	
**DNP (ETC uncoupling agent)**	Anti PD-L1	Basic research (162)	

It has been established that clinically used checkpoint blocking antibodies against CTLA-4, PD-1 and PD-L1 can partially restore T cell function (137). For example, anti-PD-L1 antibodies downregulated Akt/mTOR pathway, significantly inhibiting the glycolysis of melanoma (113) and correcting glucose restriction induced by tumors. Anti-PD-1 antibodies can directly promote the glycolysis and activation of T cells, while the blockade of CTLA-4 not only restores the glycolytic of T cells but also inhibits the function of Treg and increases the glucose content in the microenvironment (187).

Although glycolysis is an ideal target for melanoma treatment (188), immune checkpoint blockade exhibits limited ability to transform the metabolic mode of tumors (184). Drugs targeting metabolism can inhibit tumor activity and improve the ability of T cells (94) to provide an anti-tumor immune microenvironment and improve the response of patients to checkpoint therapy (113, 184). For instance, PKM2, a tumor-specific glycolytic enzyme (189), is often overexpressed in melanoma tissues (190). Since PKM2 also interacts with cytokines regulating mitochondrial fusion, it can regulate glycolysis and oxidative phosphorylation to promote the growth of tumors (191). Pharmacological targeting of PKM2 by expressing PKM2 as a tetramer or silencing its mRNA leads to inhibition of the metabolism of melanoma cells and reduced expression of PD-L1 (186). The addition of immunotherapy agents is widely thought to yield a synergistic effect.

Despite the metabolic heterogeneity induced by individual differences in melanoma patients (such as genetics, tumor vascularization, tumor stage or other factors), the dominant metabolic mode in the tumor tissue can be predicted by specific biomarkers, such as LDH (53, 103), MCT (100), PGC-1α (65, 76), etc. Importantly, therapeutic efficacy can be significantly improved by combining metabolic regulators with immunotherapy based on such biomarkers.

### 4.1 Immune checkpoint therapy with glycolysis inhibitors

It has been reported that the anti-CTLA-4 monoclonal antibody is not effective in melanoma with high glycolytic flux. However, in melanoma with glycolytic deficiency, blockade of CTLA-4 promotes infiltration and metabolic adaptation of immune cells, increases peripheral Tregs and induces its production of IFN-γ, ultimately improving the immune effect. Therefore, inhibiting tumor glycolysis in melanoma with high glycolytic flux is necessary to promote the anti-CTLA-4 effect (192–194).

Interestingly, lactic acid in melanoma is an immunosuppressive metabolite (195). It is widely thought that since Treg expresses more PD-1 than CTL, an anti-PD-1 monoclonal antibody in the environment with high lactic acid concentration will preferentially increase the flux of Treg glycolysis and enhance the immunosuppressive effect of Treg cells (16, 184). Therefore, inhibiting lactate secretion in melanoma with high glycolytic flux can improve the efficacy of immune checkpoint therapy.

Overwhelming evidence suggests that the monocarboxylic acid transporters represent a promising therapeutic target (196, 197). MCT1/2 inhibitors targeting lactate transporters are currently under investigation in clinical trials (NCT01791595). *In vitro* studies substantiated that diclofenac enhanced the killing of melanoma cells by T cells induced by anti-PD-1 and anti-CTLA-4 monoclonal antibodies (184). Diclofenac is a monocarboxylic acid salt that can directly inhibit MCT function and has recently been used as an effective inhibitor of MCT1 and MCT4. It was found that diclofenac could inhibit glycolysis in melanoma by reducing lactate transport. Although it could inhibit glycolysis in T cells (7), the secretion of IFN-γ in T cells was not blocked, possibly because T cells changed their energy metabolism mode to mitochondrial oxidation (184, 198).

LDH is an important marker in patients with advanced melanoma. Among malignant melanoma patients receiving PD-1 blockade therapy, patients with high LDHA expression show more significant ICB resistance and shorter progression-free survival (PFS) (117). Inhibition of LDH induced by oxalate and dichloroacetic acid can inhibit the growth of melanoma (96), and inhibition of LDHA improves the efficacy of anti-PD-1 treatment since the downregulation of tumor LDHA inhibits lactate production and saves glucose, and then improves the tumor microenvironment, resulting in increased T cell infiltration. The downregulation of LDHA limits the function of Tregs and contributes to a sustainable anti-tumor response during anti-CTLA-4 treatment (183). Therefore, LDH inhibition can improve the function of T cells under the immunosuppressive tumor microenvironment, making LDH an ideal target for the treatment of melanoma (52).

Inhibition of glycolysis may inhibit the activity of T cells. Since T cells cannot absorb and utilize lactate, inhibiting glycolysis enables targeting melanoma cells based on the metabolic difference between tumor and T cells.

### 4.2 Immune checkpoint therapy with OXPHOS regulator

In melanoma cells with oxidative phosphorylation as the dominant metabolic mode, the highly immunosuppressive microenvironment and the ability of mitochondria to utilize lactate caused by hypoxia account for the poor efficacy of immunotherapy in this type of melanoma patients

The OXPHOS inhibitor metformin can reportedly alleviate the hypoxic microenvironment, which is highly harmful to the function of effector CD8+ T cells (199), and then reactivate the exhausted CD8+ T cells in melanoma (200). Moreover, pharmacological inhibition against OXPHOS cannot impair the function of effector T cells (57). Metformin alone can make the tumor regress, and metformin combined with PD-1 monoclonal antibody exhibit synergistic effects during the treatment of melanoma (185, 199, 201).

It has been established that the expression of PD-1 leads to an increase in the oxidative respiratory flux of T cells, while the ROS produced by cell respiration promotes T cell apoptosis (7, 57). Current evidence suggests that anti-PD-1/PD-L1 therapy decreases the mitochondrial respiratory flux of some T cells and inhibits the mitochondrial function of T cells, although it reduces the generation of ROS (202). Intriguingly, electron transport chain (ETC) uncoupling agents can increase the production of mitochondrial ROS and enhance mitochondrial activity through the feedback mechanisms of mild mitochondrial damage (203). The joint use of ETC uncoupling agents and PD-1 antibodies can activate mTOR and AMPK pathways. Although these two pathways are antagonistic, they yield a synergistic anti-tumor effect (162). Moreover, mTOR activity can determine whether T cells transform into cells with effector or memory phenotypes after TCR stimulation and cell division (204, 205). Furthermore, low mTORC1 expression cells showed higher mitochondrial content, higher SRC (standby respiratory capacity) and more anti-apoptotic molecules, while the high mTORC1 expression showed increased glycolysis and expression of effector molecules. The AMPK pathway can act as a regulator of mTOR signaling, inhibiting glycolysis and promoting oxidative metabolism. Direct blockade of glycolysis allows T cells to preferentially migrate to lymphoid tissues and infiltrate tumors (119, 154, 157, 206). Compared with PD-1 blockade alone, the joint use of PD-1 blockade and mTOR or AMPK activation can synergistically contribute to longer patient survival (7, 162, 207). This method harnesses the effect of PD-1 monoclonal antibodies to simultaneously enhance the function of effector and memory T cells in the tumor and increase anti-tumor activity. In T cells with activated mTOR and AMPK, PGC-1α is also activated as a downstream factor, indicating that the activation of PGC-1α may also play an anti-tumor role (162).

Interestingly, Zhang et al. altered the metabolic mode of CD8+ T cells from glycolysis to mitochondrial oxidation through activating peroxisome proliferator-activated receptors α (PPAR- α) and promoting fatty acid oxidation metabolism in low glucose and the hypoxic tumor microenvironment in mice melanoma model (162). It not only delays the depletion of T cells but also maintains its anti-tumor function and promotes the formation of memory CD8+ T cells (208). In addition, OXPHOS induction of TIL can improve the efficacy of PD-1 blockade therapy in mice mode (162, 209). Etomoxir can block ROS produced by the fatty acid oxidation pathway while retaining ROS from other internal sources, thus maintaining ROS physiological levels to activate T cells and reducing apoptosis (210).

In conclusion, increasing glycolytic or OXPHOS brings different advantages for T cells. Increasing glycolysis to promote immune effects is a better choice when glucose or glutamine is abundant in the tumor microenvironment. In the absence of glucose, it is more suitable to increase OXPHOS to maintain the number of immune cells.

## 5 Discussion

Energy metabolism in melanoma is influenced by melanoma gene mutations, oxygen, lactate, and degree of vascularization. The TME in advanced melanoma contains both hypoxic and normoxia regions, which simultaneously elevated both glycolysis and OXPHOS. This heterogenous metabolic pattern not only suppresses immune function but also leading to poor immunotherapy efficacy. Importantly, the main metabolic pattern of melanoma can be predicted by relevant proteins. Clinical data are available on metabolism-related protein levels as biomarkers to determine the metabolic status of tumors, such as LDH, MCT1/4, PGC-1α, etc. Therefore, harnessing metabolic modulators to improve the tumor microenvironment based on the predicted results of metabolic modality will improve the efficacy of immunotherapy.

However, the pathways affecting heterogeneous metabolism in melanoma have been largely understudied, and metabolic heterogeneity in the tumor microenvironment cannot be accurately quantified. Moreover, much uncertainty surrounds screening biomarkers associated with tumor metabolic heterogeneity, such as the location of metastases and individual patient differences. More studies with large sample sizes are warranted in the future to validate metabolism-related biomarkers and explore the mechanisms of biomarker effects on melanoma cells.

## Author contributions

XZ and ZT wrote the draft and revised it. FM, HH, LB, ZC, QZ designed the article and helped revise it. All authors contributed to the article and approved the submitted version. XZ and ZT contributed equally to this work.
